# Letter to the editors regarding the paper: Prognostic factors in HIV-positive patients with non-Hodgkin lymphoma: a Peruvian experience

**DOI:** 10.1186/s13027-018-0213-6

**Published:** 2018-12-11

**Authors:** Mirtha P. Rivera-Castillo, Diana Quispe-Pineda, Aldo J. Lucchetti

**Affiliations:** 1 Escuela de Medicina, Facultad de Ciencias de la Salud, Universidad Peruana de Ciencias Aplicadas, 2 Alameda San Marcos Avenue, Chorrillos, 15067 Lima, Peru; 2 Servicio de Infectología, Hospital Nacional Arzobispo Loayza, 848 Alfonso Ugarte Avenue, Cercado de Lima, 15082 Lima, Peru

**Keywords:** HIV, Non-Hodgkin lymphoma, AIDS-related Kaposi sarcoma, Herpesvirus 8

## Abstract

Non-Hodgkin Lymphoma (NHL) is a neoplasm associated with a group of malignancies called AIDs-Defining Malignancies (ADMs) in Human-Immunodeficiency Virus (HIV) -patients. Similar to the case of NHL in Latin America, particularly in Peru, the amount of research done on others ADMs is limited, especially in the case of Kaposi’s Sarcoma (KS). Prior investigations have talked about the great potential risk that represents this illness in latin american population, but topics as prognosis factors are yet to be well defined. In this letter, we address the importance of investigation in this area and include previously reported data that may enlighten the current national standpoint.

## Main text

We have read with great interest the article previously presented in this journal regarding the prognosis factors in Human-Immunodeficiency Virus (HIV) -patients with non-Hodgkin Lymphoma (NHL) [1]. This study encouraged us to elaborate on the importance of investigation about this type of malignancies, including another relevant neoplasm such as Kaposi’s Sarcoma (KS).

As previously mentioned by Cuellar et al., the number of researches done in Latin American countries regarding AIDs-Defining Malignancies (ADMs) is marginal [1]. Likewise, the author affirmed that this is the first study of its sort made in Peruvian population and the first concerning survival in NHL in Latin American population, defining relevant prognosis factors such as being ART-naïve, having low albumin levels, and the tumor staging [1]. As evidence, the last research regarding NHL and its prognostic factors was made in 2009 by Rezende et al. [2]; there were not following investigations that comprised these topics until Cuellar [1]. This observation is relevant because it provides a perspective of the research status in other illnesses such as KS, where there is no precedent study respecting survival or prognostic factors. Therefore, the lack of investigation in this area appears to affect NHL and other ADMs.

Delving in KS, it is a neoplasm related with a viral etiology caused by both Herpesvirus type 8 (HHV-8) and HIV [3–5]. In Peru, according to prior investigations, the incidence of HHV-8 on mixed population is relatively high; with a report by Mohanna et al. who finds a prevalence of 56,25% in a study with 128 blood donors non-HIV carriers [6–8]. With no actual published information, we can only infer that Peru may have a high prevalence of patients that may be at risk of developing SK, with the presence of its prognostic factors, or currently are manifesting the disease by the information stated beforehand.

It has been reported that prognostic factors in NHL are similar to KS, probably because of their relationship with HIV [4]. In spite of this information, it has been established that the prognostic factors in KS are not well defined; stating that even CD4 count, crucial information in an average HIV-patient, does not represent a significant factor in the prognosis of KS [9]. This may occur because of the changes in treatment, the introduction of Highly Active Antiretroviral Therapy (HAART), and the lack of survival research [9].

Finally, we held in high regards your research in this kind of population and subjects, both which tends have investigations that do not bring much data. Because of the importance of your study, we considered important to broaden this kind of interest in the same area, so we can contribute bringing more information to the literature through longitudinal studies, like prospective or retrospective cohort studies.

## Abbreviations

ADMs: AIDs-Defining Malignancies
HAART: Highly Active Antiretroviral Therapy
HHV-8: Herpesvirus type 8
HIV: Human-Immunodeficiency Virus
KS: Kaposi’s Sarcoma
NHL: Non-Hodgkin Lymphoma
